# Assessment Tools of Patient Competences: The Spanish Version of the R-NPQ and Three Practical Cases in Women with Breast Cancer and Persistent Pain

**DOI:** 10.3390/ijerph18094463

**Published:** 2021-04-22

**Authors:** María Torres-Lacomba, Beatriz Navarro-Brazález, Javier Bailón-Cerezo, Fernando Vergara-Pérez, Irene de la Rosa-Díaz, Virginia Prieto-Gómez

**Affiliations:** 1 Physiotherapy in Women’s Health (FPSM) Research Group, Physiotherapy Department, Faculty of Medicine and Health Sciences, University of Alcalá, 28801 Madrid, Spain; maria.torres@uah.es (M.T.-L.); b.navarro@uah.es (B.N.-B.); fernando.vergara@uah.es (F.V.-P.); irenedelarosadiaz@gmail.com (I.d.l.R.-D.); v.prieto@uah.es (V.P.-G.); 2 Centro Superior de Estudios Universitarios La Salle, Department of Physical Therapy, Universidad Autónoma de Madrid, 28049 Madrid, Spain

**Keywords:** breast cancer, patient competence assessment, competence assessment tools, pain, patient education, revised neurophysiology of pain questionnaire (R-NPQ), Spanish validation

## Abstract

Persistent pain following treatment for breast cancer (PPBCT) is a prevalent and complex clinical issue. Education together with physiotherapy have been shown to lessen pain and disability in chronic pain. Although the evaluation of the patient’s competences is a major part of the educational program, the published educational programs rarely describe the tools used to assess competences, especially regarding those related to decision-making and problem-solving. The aim of this study was to provide two competences assessment tools: the cross-cultural adaptation and validation of the Spanish version of the Revised Neurophysiology of Pain Questionnaire (R-NPQ) and practical cases of women with PPBCT. The Spanish cross-cultural adaptation was conducted following recognized criteria. Measurement properties testing included an analysis of construct validity (known-groups approach), reliability (internal consistency and test-retest reliability), responsiveness, interpretability, and feasibility. To promote a tool that would allow evaluation of the educational program competences, a group of experts developed three cases extracted from real contexts by means of an iterative process. A total of 80 women with PPBCT (mean age 56 years) and 81 physiotherapy students (mean age 20 years) participated in the measurement properties analysis. The three developed cases were presented to the same 80 women with PPBCT before and after the educational program. As we expected, students showed a significantly higher score (*p* < 0.001) than did women with PPBCT in the R-NPQ questionnaire, with a large effect size (d = 2.49), demonstrating good construct validity. The Cronbach alpha was 0.90 (95% CI, 0.87–0.92) and the intraclass correlation coefficient was 0.82 (95% CI, 0.73–0.88). A large effect size (5.2) was found, as we expected, between baseline and post-treatment scores, suggesting adequate responsiveness. In addition, identifying and analyzing, decision making, communicating needs, knowing how to manage, and problem-solving skills were evaluated through the three practical cases. Most women (88.75%) reached the highest level in the assessment rubric of the proposed practical cases. The Spanish R-NPQ is a comprehensible, valid, reliable, and responsive tool for Spanish women with PPBCT. The practical cases are a useful competence assessment tool and were well accepted by women with PPBCT. Further studies are needed to investigate more competence assessment tools and to investigate whether the achievement of different levels of competences has an effect on health behaviors.

## 1. Introduction

Breast cancer (BC) survival rates have increased over time because of improvements in early detection and treatment. In the 28 European Union countries the five-year survival ranges from 79% to 93% [1]. However, upper body persistent pain following treatment for breast cancer (PPBCT) is a potential complication affecting 25 to 60% of patients, fluctuating considerably over time [2], and has been associated with reduced functional status, well-being, and quality of life [2,3,4,5]. A recent systematic review concluded, based on 177 studies (135,437 patients), that PPBCT remains a prevalent and complex clinical issue ranging from 21.8% to 29.8% depending on whether it is post-surgery, post-radiotherapy, or post various combinations of BC treatment [5]. Many patients with PPBCT do not achieve proper pain control [6]. BC treatments can lead to both nociceptive and neuropathic pain in the upper body, suggesting the contributory role of treatments to [4,7] PPBCT, along with other factors like younger age, more invasive surgery, adjuvant therapy, and psychosocial factors [3]. PPBCT has also been associated with pain catastrophizing, breast cancer worries, and emotional distress [8]. BC survivors who are experiencing PPBCT are treated with a variety pharmacological and non-pharmacological options [9]. Among the non-pharmacological options, physiotherapy [10,11,12,13,14,15], education [16], and psychosocial [17] support for cancer patients and their families have become the standard multimodal approach of BC care.

Regarding patient education, a patient-centered approach to health care has shown increased patient satisfaction and better health outcomes, especially in chronic diseases [18]. This approach was outlined by WHO in 1998 as therapeutic patient education (TPE). According to the WHO, TPE “is designed therefore to train patients in the skills of self-managing or adapting treatment to their particular chronic disease, and in coping processes and skills. […..] Its main purpose is to produce a therapeutic effect additional to that of all other interventions (pharmacological, physical therapy, etc.)” [sic] [19]. Related to chronic pain, we found enough evidence suggesting that a proper patient education about pain neurophysiology could improve the perception of pain, pain tolerance, and health status [8,19]. However, few studies can be found about women with PPBCT, only a controlled trial [20] and three study protocols [21,22,23], but neither competences of the educational program nor how they knew if the patients had achieved them were described in any of the studies. In any TPE program, it can be expected that patients and their families master a variety of competences. In a broad sense of the word, competence is defined as the application of knowledge, skills, and behaviors used by patients and their families to transform their health status [24]. This broad vision takes shape in the context of patient learning in TPE programs. In this context of patient learning, the evaluated competences reflect a pedagogical transformation and are understood as an evolutionary and progressive process of acquisition of knowledge, cognitive and metacognitive, interpersonal skills, practices, and ethical values that can be improved and thoroughly evaluated by means of a continuous process where different learning and assessment processes and tools can be used to develop and specify levels of achievement of competence [24,25,26]. Within this context, a patient’s competences include identification, analysis, and interpretation of clinical signs and scenarios; problem-solving; making informed decisions, the ability to provide an overview of complaints, to discuss specific health goals and challenges, to manage the information and resources, and to build relationships with providers; etc. [19,24]. These competences must therefore be evaluated. Evaluation in the pedagogical domain ensures that learning has taken place among patients [19]. Unfortunately, no competence assessment tools are found in educational programs, at least as regards competences related to decision-making and problem solving. Regarding chronic pain, all educational programs on pain must include pain neurophysiology knowledge. Moseley developed the Neurophysiology of Pain Questionnaire (NPQ) in 2003, its revision resulted in a new version, the Revised Neurophysiology of Pain Questionnaire (R-NPQ). Despite some limitations, it seems to be a useful tool to measure patient knowledge levels about pain neurophysiology [27]. However, it does not assess how patients can transfer this knowledge to real-life situations or to their specific situation to make decisions or solve problems. Therefore, this research aimed to translate and validate to the Spanish language the R-NPQ for women with PPBCT as well as proposing practical cases as a competence assessment tool where patients have active participation with the purpose of offering detailed and clear tools for evaluating different levels of competences of an educational program for women with PPBCT.

## 2. Materials and Methods

### 2.1. R-NPQ Spanish Cultural Adaptation and Measurement Properties Testing

A cross-sectional observational study was conducted from February 2018 to February 2020. This study (2013/015/2010624) was approved by the local Hospital’s Clinical Research Ethics Committee in Alcalá de Henares (Madrid, Spain). The “Strengthening the Reporting of Observational studies in Epidemiology” (STROBE) guidelines recommendations were followed.

This study was conducted in two steps: (1) cross-cultural translating of the English R-NPQ into a Spanish version; and (2) testing of the measurement properties of the R-NPQ Spanish version.

We would like to emphasize to readers that the R-NPQ has been used by this research team for years, and that the R-NPQ version presented here is the one provided by bodyinmind.org web, currently inactive. This version can currently be found on the Yumpu digital platform (https://www.yumpu.com/en/document/read/40272202/revised-neurophysiology-of-pain-questionnaire-body-in-mind) and consists of 13 statements about pain instead of the 12 that are found in a few cross-cultural validations of the R-NPQ [28,29,30]. This does not prevent the use of this Spanish version (see Appendix A), which highlights the additional statement so that the Spanish R-NPQ of the 12 statements can also be used [27].

#### 2.1.1. Step 1: Translation and Cultural Adaptation

The cross-cultural adaptation of the R-NPQ was conducted in three phases according to the International Society for Pharmacoeconomics and Outcomes Research (ISPOR) Task Force for Translation and Cultural Adaptation [31]. In the first phase, the English R-NPQ was translated and culturally adapted independently by two English–Spanish translators (native Spanish speakers). The research team, including physiotherapists with previous experience in pain neuroscience education, agreed to the synthesis of the Spanish translations.

In the second phase, two Spanish–English translators (native English speakers) did the back-translation (blind to the original version of the R-NPQ). Following, a review by an Expert Committee (one methodologist, two physiotherapists who are experts in pain neurophysiology, and one Spanish language professional) a prefinal version of the R-NPQ Spanish version was obtained.

In the third phase, to test comprehensibility, the R-NPQ prefinal Spanish version was provided to 20 native Spanish-speaking women who fulfilled the inclusion criteria (patients or expert group criteria). They completed it and were questioned about any difficulties encountered in completing the questionnaire. Following the interview process, the expert committee proposed the final R-NPQ Spanish version (see Appendix A).

#### 2.1.2. Step 2: Measurement Properties Testing

##### Participants and Procedure

Spanish-speaking women treated for BC with PPBCT were recruited at the Women’s Health Research Unit of the University of Alcalá (Madrid, Spain) and included in the study. Women who met the following inclusion criteria were eligible for the study: women aged ≥ 18 years; PPBCT (>3 months); maladaptive pain cognitions, illness perceptions, or coping strategies measured with the Pain Catastrophizing Scale; able to read and understand Spanish; without cognitive problems to complete the questionnaire. Women with a history of specific pain neurophysiology education courses within the last 12 months were excluded.

To study the construct validity (discriminative validity) of the R-NPQ-Spanish version, a group of Spanish-speaking students in the final year of a Degree in Physiotherapy at the University of Alcalá (Madrid, Spain), trained in pain neurophysiology, was also included.

Written informed consent was obtained from all the participants.

Data were collected from both groups (women with PPCBT group: sociodemographic and clinical data; Control group: sociodemographic data). Next, participants completed the R-NPQ Spanish version. One week later, 25 women with PPCBT were asked to fill in the R-NPQ Spanish version again to assess the test-retest reliability. Then, they were included in a pain therapeutic education program (Table 1) based on the recommendations of Nijs et al. [12,32]. After completing the pain TPE program, all women completed the R-NPQ Spanish version a third time to evaluate responsiveness. Average time was recorded for the first administration of the questionnaire.

##### Questionnaires

The general questionnaire included questions about gender (only Control Group), age, duration and location of pain, individual’s pain experience and catastrophic thinking related to pain by the catastrophizing pain scale Spanish version [33] (only PPCT Group), education level, learning preferences, and work status.

The R-NPQ Spanish version (Body in Mind Research Group, University of South Australia) consists of 13 (True/False/I do not know) pain statements. The overall R-NPQ Spanish version score ranges from 0 to 13 (sum of all correct items) or is expressed as a percentage.

#### 2.1.3. Data Analysis

Statistical analyses were conducted using SPSS Version 23.0 (IBM Corp). Descriptive statistics were calculated using the arithmetic mean and SD as indices of central tendency and dispersion for the quantitative variables or using the median and interquartile ranges when wide dispersions conditioned the interpretation of the variable. Absolute and relative percentage frequencies were used for the categorical variables. The inferential analysis was estimated with a 95% CI, considering a *p*-value < 0.05 as statistically significant.

Sample size was based on the general recommendations by Altman et al. and Terwee [34] who stated that at least 50 subjects would be recommended for the assessment of measurement as well as on the Bryant and Yarnold [35] recommendations related to the subjects-to-variables ratio that should be no lower than five.

Measurement properties testing was developed following the recommendations of the consensus-based standards for the selection of health measurement instruments (COSMIN) [36].

*Interpretability and feasibility:* Score distribution, percentage of missing items, and floor and ceiling effects (>15% of the sample with minimum or maximum scores, respectively) [31] were calculated. Completion time in the physiotherapy student group was calculated.

*Validity:* A known-groups approach (also known as discriminative validity) was selected to explore the construct validity of the R-NPQ questionnaire. Physiotherapy students were expected to score statistically significantly higher than patients, with a large effect size in mean differences. Student´s t test was employed for this comparison and Cohen´s d for effect size calculation.

*Reliability:* Cronbach´s α was calculated as an estimator of internal consistency, assuming a value ≥0.7 as acceptable [36]. This analysis was performed for patients (baseline), students, and the entire sample.

The test-retest reliability for the total scores was calculated with a two-way random effect, single measures, and absolute agreement intraclass correlation coefficient (ICC) model [31,36], assuming a value ≥0.7 as acceptable [37]. Standard error of measurement (SEM) and smallest detectable change (SDC) were calculated from the test-retest reliability study. The SEM was calculated as $\mathrm{SD}\;x\sqrt{1-\mathrm{ICC}}$, where SD was the standard deviation of the mean of all observed scores and ICC was the reliability estimator reported. Smallest detectable change (SDC) was calculated as $\mathrm{SEM}\;x\;1.96\;x\sqrt{2}$ for an individual level and as $\mathrm{SEM}\;x\;1.96\;x\sqrt{2}/n$ for a group level [37].

*Responsiveness:* A large effect of the education intervention on the construct measured by the R-NPQ questionnaire was expected, based on previous findings [38,39]. For this purpose, effect size (mean change score/SD baseline) and standardized response mean (SRM) (mean change score/SD change score) [37] for the difference between post-intervention and baseline score in the R-NPQ questionnaire were calculated. Values of 0.20–0.49 were considered small, 0.50–0.79 medium, and of 0.8–1.0 large effects [40].

### 2.2. Development of the Practical Cases

Our team of physiotherapists, assisted by an educational evaluation and assessment specialist, developed three cases extracted from real contexts (see Appendix B) by means of an iterative process. They were related to the competences to be assessed (see Appendix B), as well as to the knowledge to be mobilized during the discussion of the practical cases with women with PPBCT. Finally, practical cases were tested with 80 women with PPBCT who participated in the pain TPE program to evaluate the understanding and usefulness of the practical cases. The physiotherapist urged the patient to identify and analyze the case data, detect the perpetuating and triggering factors of the problem, explain them according to the problem, propose solutions, etc. In addition, to assess the level of competence achieved in solving the cases, the rubric of evaluation of practical cases described elsewhere was used [41].

The two evaluation tools presented here reflect the patient learning achieved as part of this TPE and therefore aim to evaluate the patient’s competences (see Appendix B) and the elements constitutive of these competences [24,25].

## 3. Results

### 3.1. R-NPQ Spanish Cultural Adaptation and Measurement Properties Testing

#### 3.1.1. Translation and Cultural Adaptation

Translation and backtranslation were conducted without any disagreement between translators. Comprehensibility analysis with 20 women with PPCBT revealed some misunderstanding regarding item number 9. Translation issues were also suggested taking into account the contributions of participants and the expert committee produced a Spanish final version.

#### 3.1.2. Measurement Properties Testing

Eighty women with PPBCT and 81 physiotherapy students (45 females and 36 males) were included in the present study. Descriptive data of the sample are shown in Table 2. All students received 2 h of training in pain neurophysiology, while all women completed the educational program described in Table 1. Concerning feasibility, the average time for questionnaire completion was 3.45 (±1.6) min.

*Interpretability and feasibility*: Score distribution on the patient and physiotherapy student groups are shown in Table 3. There were no missing items. Forty-six patients (57.5%) obtained the minimum score at baseline, showing floor effects.

*Construct validity:* The hypothesis regarding score differences was confirmed, with patients performing significantly worse (*p* < 0.001) on the R-NPQ questionnaire than the physiotherapy students did, with a large effect size (*d =* 2.49) for differences between groups.

*Reliability:* Cronbach’s alpha showed good internal consistency for the R-NPQ questionnaire, with a value of 0.90 (0.87–0.92) when the entire sample was analyzed. For patients, Cronbach’s alpha was 0.82 (0.75–0.87) and for students 0.72 (0.62–0.80), also showing acceptable values.

In the test-retest reliability, the ICC also revealed a good test-retest validity. Data obtained through test-retest analysis are presented in Table 4.

*Responsiveness:* As was expected, a large effect size was found between the final and baseline scores of the intervention group (Table 4), indicating that the questionnaire reflects the large changes found in previous studies with educational intervention.

### 3.2. Practical Cases Proposal

Descriptions of the practical cases and the related competences are shown in Appendix B. Competences, knowledge and behaviors, and achievement assessment are described. The rubric for evaluating practical cases can be found elsewhere [41]. The R-NPQ assesses knowledge related to the neurophysiology of pain, also allowing for the detection of beliefs regarding pain; however, it does not evaluate knowledge transference to possible real situations. Therefore the practical cases were proposed as a tool for evaluating knowledge transference to real-world scenarios, in other words, the ability to evaluate competences related to identifying and analyzing, decision making, communicating needs, knowing how to manage, and problem solving [24]. The three developed cases were presented to the women with PPBCT before and after the TPE program. In the post-educational evaluation, 71 (88.75%) women reached the highest level of “ Gold Medal”, and 9 (11.25%) reached the level of “Well Done” in the assessment rubric of the proposed practical cases [41]. Most of the women found the cases playful, interactive, and participatory. They also expressed that these cases allowed them to relate knowledge and skills from the educational program. Moreover, they allowed them to discuss doubts with the physiotherapist, as it was a face-to-face evaluation.

## 4. Discussion

A patient-centered approach to health care by TPE has shown to improve the perception of pain, pain tolerance, and health status [8,19]. The development of an TPE program should include the assessment of competences from various perspectives and tools [42]. In this study, the validation in Spanish by women with PPBCT of the R-NPQ and practical cases were developed as tools for evaluating competences of an TPE program for women with PPBCT.

To our knowledge, this is the first study that details the competences, the educational program, and, the evaluation of competences tested in women with persistent pain after breast cancer treatment for the purpose of verifying the pedagogical transformation of the patient. 

An equivalent and comprehensible Spanish version of the R-NPQ questionnaire was achieved after the cross-cultural adaptation process. Measurement properties testing of this version provided evidence of its construct validity, internal consistency, test-retest reliability, and responsiveness.

Comprehensibility, comprehensiveness, and content relevance are the components of content validity [31]. Relevance and comprehensiveness of the NPQ questionnaire is supported by the original development study [27]. In addition, the present study proves the comprehensibility of the instrument in the Spanish population.

While the questionnaire did not show ceiling effects in patients nor physiotherapy students, suggesting its ability to capture improvements in pain neuroscience knowledge, floor effects were found between patients when tested before treatment. The French version of the R-NPQ questionnaire also reported floor and ceiling effects, but this floor effect was not found [28]. A possible explanation is the difference in baseline knowledge between the two samples. Their sample of patients with spinal pain showed a mean (SD) score of 5.6 (1.8), while in our study it was 1.18 (2.07), even though our version scores was over 13 instead of 12 statements. Floor effects imply a difficulty in capturing a deterioration in the construct measured by the questionnaire and to distinguish between patients with low knowledge [34]. Thus, this should be considered when using this Spanish version in clinical practice and research.

Regarding the feasibility of the questionnaire, a short time of completion was found. This situation, together with the ease of completion and correction, makes this questionnaire a tool of low burden for patients and clinicians/researchers.

Construct validity was tested using a known-groups or discriminative validity approach, as was done in French, German, and Brazilian Portuguese adaptations [28,29,30]. Results were consistent across all adaptations, providing clear evidence for discriminant validity. Internal consistency results (α = 0.9) revealed higher values than in French (α = 0.3), German (α = 0.52), and Brazilian Portuguese (α = 0.63) adaptations, whilst test-retest reliability (ICC = 0.82) was in line with results of the German adaptation (ICC = 0.88). While test-retest is related to the reliability of the total score of the questionnaire, internal consistency means that items are interrelated, and they measure consistently [36]. The slight differences in the items between our version and other adaptations could explain our better results.

Previous adaptations did not test responsiveness, but our study suggest a good capacity to detect changes in the construct measured. We relied on a hypothesis testing approach, suggested by the COSMIN initiative. Once we knew from previous studies the positive effects of an educational intervention, we expected to find a positive change in the score of the R-NPQ questionnaire after this kind of intervention [27]. As occurs with construct validity, different approaches can be adopted to assess this property [36], and future studies should corroborate our results. The fact that women after the TPE scored higher than students at baseline can be explained by the different education approaches employed in each group. While the contents of both programs were developed according to the recommendations of Nijs et al. [12,32], students only received a 2 h group training while women received an individualized program detailed in Table 1.

Any TPE program must carry out an assessment focused on the patient’s competences [19,24]. Most of the educational programs published did not deal with participants’ experiences when tools assessing competences were used. This study presents an experience with the assessment tool of practical cases to assess the acquisition of skills related to identification, analysis, and interpretation of clinical signs and scenarios; problem-solving; and making informed decisions in women with PPBCT.

We developed a program with competence assessment combining a behavioral vision based on the culture of evaluation of results, guided by hetero-evaluation processes and a more constructivist vision with active participation of the patients in which reflective processes are carried out in collaboration with others [42,43]. This program also includes the practical cases for patients as authentic activities [44,45] and tools for their assessment. Practical cases for patients are a genuine learning approach that lead to an overall competence evaluation that focuses more on competence than simply on knowledge [24,25], and they were recognized by women with PPBCT as playful, participatory, iterative, and as an opportunity to integrate knowledge and skills. These encouraging results, together with the opinions of the women with PPBCT on the cases turned out to be a promising educational approach.

Even though most of the women with PPBCT reached the highest level of knowledge in the R-NPQ, as well as the highest level of acquisition of competences in the rubric of evaluation of practical cases, further studies are needed to investigate whether the achievement of competences has an effect on health behaviors.

## 5. Conclusions

The Spanish R-NPQ is a comprehensible, valid, reliable, feasible, and responsive-to-change tool for assessing pain neurophysiology knowledge in Spanish women with PPBCT.

Practical cases are a useful competence assessment tool related to identification, analysis, and interpretation of clinical signs and scenarios; problem-solving; and making informed decisions. These competence assessment tools are well accepted by women with PPBCT.

Further studies are needed to investigate and propose more competence assessment tools and to investigate whether the achievement of different levels of competency has an effect on health behaviors.

## Figures and Tables

**Table 1:** Therapeutic education program for breast cancer survivors with persistent pain.

Sessions	Topics	Contents	Learning Tools	Activities	Assessment Tools
1 (45 min one-on-one session)	- What do I know about my pain? - Could my pain be related to my breast cancer treatments? - How do I live with my pain?	- Knowledge about why therapeutic education about health problems is important - Knowledge relates to the structure and general objectives of the pain education program	- Exploratory motivational interview - Pain catastrophizing scale - R-NPQ test	- Answer chained questions about pain features; pain experiences, perceptions, and beliefs; pain catastrophizing, kinesiophobia and fear-avoidance; pain coping strategies; movement disturbances; cancer-related fatigue; lymphoedema, social support; and knowledge about treatment options - Explore expectations and individual goals about the education program - Create a commitment document that reflects her individual goals for this program - Fulfill the Pain Catastrophizing Scale and the R-NPQ Test	- Patient/physiotherapist discussion about goals and program structure
2 (30 min one-on-one session)	Why I feel pain? Part I	- Basic knowledge about structure and function of the nervous system - Basic knowledge related to pain physiology adapted to the participant’s predominant pain mechanism (I): nociception, peripheral sensitization; acute pain vs. persistent pain	- Anatomical prints - Nervous system anatomical model - Pain knowledge slides (© FPSM Group) - *Understanding Pain in less than 5 min, and what to do about it!* Spanish Version: video - *Explain Pain* Spanish Version: book (Butler D et al., 2013)	- Identify the parts of nervous system in the anatomical prints and anatomic model - Watch the slides presentation and the video of pain - Recognize the book chapters related to the session to read at home	- A basic drawing of the main parts of nervous system is required - A table with the differences between acute pain vs. persistent pain is required
3 (30 min one-on-one session)	Why I feel pain? Part II	- Basic knowledge related to pain physiology adapted to the participant’s predominant pain mechanism (II): nociceptive pain vs. neuropathic pain vs. central sensitization; pain memories; contributing and aggravating personal factors in the pain perception (cognitive, psychological, behavioral, social factors) - Basic knowledge about the causes and evolution of pain, movement disturbances, cancer-related fatigue, and lymphoedema induced by oncological treatments	- Pain knowledge slides (© FPSM Group) - *Explain Pain* Spanish version: book (Butler D et al., 2013*)*	- Watch the slides presentation of pain - Recognize the book chapters related to the session to read at home - Identify contributing and aggravating personal factors as well as those associated to breast cancer treatment factors that determine her pain	- Pain cards game: cards with short phrases that include random pain features to associate with the different pain mechanisms - Patient/physiotherapist discussion about which contributing and aggravating factors determine her pain
4 and 5 (both one-on-one sessions and 30 min each)	What pain coping strategies can I adopt to improve my pain?	- Knowledge about individual pain mitigation and management strategies according to the paticipant’s pain contributing and aggravating factors (e.g., behavioral changes linked to kinesiophobia and fear-avoidance, negative thoughts, attention to pain and stress, among others) - Knowledge about healthy lifestyle habits: physical activity and exercise, stress management, nutrition and dietary habits, sleep, hygiene - How movement and therapeutic exercise can help overcome persistent pain, movement disturbances, and cancer-related fatigue as well as prevent and manage lymphoedema induced by oncological treatments	- Pain knowledge slides (© FPSM Group) - Motor imaginary - Physiotherapist manual and auditory feedback - 3D Vision Camera Kinect © feedback - Perceived pain and coping strategies diary - *Explain Pain* Spanish version: book (Butler D et al., 2013*)*	- Propose behavioral changes related to personal psychosocial factors (e.g., amusement, stress-relieving activities) - Complete perceived pain & coping strategies diary for 7 consecutive days (after 4 and 5 sessions) - Recognize the book chapters related to the session to read at home - Start a gradual exposure to movement by cognition-targeted exercises following a time-contingent approach	- Flow chart that outlines how she will handle her therapeutic process based on the facilitating factors and barriers identified - A diary is requested and will be analyzed later - On-site exercise movement are checked
6 (45 min one-on-one session)	What have I learned about my pain?	- Total contents of the pain educational program (session 1 to 5)	- Exploratory motivational interview - Practical cases - R-NPQ Test	- Complete the R-NPQ test again - Detect pain features, contributing and aggravating factors in pain perception, as well as the coping strategies options for every practical case	- Patient/physiotherapist discussion about R-NPQ test and rubric of practical cases

R-NPQ test: Revised-Neurophysiology Pain Questionnaire; FPSM: Research Group “Physiotherapy in Women’s Health Research Group” of Alcalá University, Spain.

**Table 2:** Characteristics of the sample.

Variables	Women with PPCBT (*n* = 80)	Physiotherapy Students (*n* = 81)
Age (years). Mean (SD)	56.04 (8.85)	20.06 (1.44)
Education level, *n* (%)	Primary education: 17 (21.3)	University degree: 81 (100)
	Secondary education: 51 (63.7)	
	Pre-university or professional education: 8 (10)	
	University degree: 4 (5)	
Duration of pain (months)	5 (8)	-
Median (IQR)		
Pain location (*n*, %)	Shoulder: 1 (1.3%)	-
	Scapula: 20 (25%)	
	Arm: 8 (10%)	
	Shoulder and scapula: 16 (20%)	
	Shoulder and arm: 1 (1.3%)	
	Scapula and arm: 9 (11.3%)	
	Shoulder, scapula, and arm: 25 (31.3%)	
Pain Catastrophizing Scale Percentile. * Mean (SD)	Rumination: 85.71 (12.12)	-
	Magnification: 94.18 (5.56)	
	Helplessness: 93.51 (6.92)	
	Total: 93.39 (7.47)	

SD: Standard Deviation. IQR: Interquartile range. PPBCT: Persistent pain following breast cancer treatments. * Higher scores above the 75th percentile indicate higher levels of pain-related anxiety.

**Table 3:** Scores and effect sizes of R-NPQ questionnaires for patient and expert groups.

Groups	Baseline Score, $\overset{-}{x}$ (SD)	Minimum Score, *n* (%)	Maximum Score, *n* (%)	Post-Intervention Score, $\overset{-}{x}$ (SD)	Effect Size (95% CI)	SRM (95% CI)
**Women with PPBCT (*n* = 80)**	1.18 (2.07)	46 (57.5)	0 (0)	11.94 (1.33)	5.2 (3.8–7.3)	4.3 (3.3–5.5)
**Physiotherapy students (*n* = 81)**	7.37 (2.84)	2 (2.5)	0 (0)	—	—	—

$\overset{-}{x}$ (SD): Mean (standard deviation); CI: Confidence interval; SRM: Standardized response mean; —: Not applicable; PPBCT: Persistent pain following breast cancer treatments.

**Table 4:** Data derived from test-retest reliability analysis.

	Test, $\overset{-}{x}$ (SD)	Retest, $\overset{-}{x}$ (SD)	SEM	SDC_ind_	SDC_group_	ICC (95%CI)
**Women with PPBCT (*n* = 25)**	1.18 (2.07)	11.9 (1.49)	0.57	1.57	0.42	0.82 (0.73–0.88)

$\overset{-}{x}$ (SD): Mean (standard deviation); SEM: Standard error of the measurement; SDCind: Smallest detectable change (individual level); SDCgroup: Smallest detectable change (group level); ICC: Intraclass correlation coefficient; CI: Confidence interval;PPBCT: Persistent pain following breast cancer treatments.

## Data Availability

The data presented in this study are available from the corresponding author upon reasonable request.

## Appendix A

Ítems	**Versión Española del Cuestionario sobre la Neurofisiología del Dolor**	**V**	**F**	**NS**
1	Es posible sentir dolor y no darse cuenta.			
2	Cuando una parte de su cuerpo está lesionada, unos receptores especiales del dolor transmiten el mensaje de dolor al cerebro.			
3	El dolor sólo se produce cuando usted se lesiona o corre el riesgo de lesionarse.			
4	Cuando usted se lesiona, unos receptores espe-ciales transmiten el mensaje de ‘peligro’ a su mé-dula espinal.			
5	Unos nervios especiales en su médula espinal transmiten mensajes de ‘peligro’ a su cerebro.			
6	Los nervios se adaptan aumentando su nivel de excitación en reposo.			
7	El dolor crónico indica que una lesión no se ha curado completamente.			
8	Las lesiones más graves siempre causan dolor más intenso.			
9	Los nervios se adaptan haciendo que los canales iónicos permanezcan abiertos más tiempo. *			
10	Las neuronas descendentes son siempre inhibito-rias.			
11	El dolor se produce cuando usted se lesiona.			
12	Cuando usted se lesiona, el entorno en el que usted se encuentra no afectará a la cantidad de dolor que experimente, siempre y cuando la lesión sea exactamente la misma.			
13	El cerebro decide cuando usted experimentará dolor.			
* Statement 9 is not included in the few currently existing cross-cultural validations of the R-NPQ.				

Respuestas:

**Ítems**	**Respuestas al Cuestionario sobre la Neurofisiología del Dolor**	**V**	**F**	**NS**
1	Es posible sentir dolor y no darse cuenta.		#	
2	Cuando una parte de su cuerpo está lesionada, unos receptores especiales del dolor transmiten el mensaje de dolor al cerebro.		#	
3	El dolor sólo se produce cuando usted se lesiona o corre el riesgo de lesionarse.		#	
4	Cuando usted se lesiona, unos receptores espe-ciales transmiten el mensaje de ‘peligro’ a su mé-dula espinal.	#		
5	Unos nervios especiales en su médula espinal transmiten mensajes de ‘peligro’ a su cerebro.	#		
6	Los nervios se adaptan aumentando su nivel de excitación en reposo.	#		
7	El dolor crónico indica que una lesión no se ha curado completamente.		#	
8	Las lesiones más graves siempre causan dolor más intenso.		#	
9	Los nervios se adaptan haciendo que los canales iónicos permanezcan abiertos más tiempo.	#		
10	Las neuronas descendentes son siempre inhibito-rias.		#	
11	El dolor se produce cuando usted se lesiona.		#	
12	Cuando usted se lesiona, el entorno en el que usted se encuentra no afectará a la cantidad de dolor que experimente, siempre y cuando la lesión sea exactamente la misma.		#	
13	El cerebro decide cuando usted experimentará dolor.	#		

## Appendix B

***Real practical case 1:*** *Soraya has been in bed for 5 days and she feels frustrated and depressed. She feels things are not going well since chemotherapy treatment finished. She suffers from ongoing-widespread pain. She is wondering all the time why she suffers from such pain “¿will it be due to any serious disease? And being constantly aware of any painful symptoms and always vigilant of how the body feels. She has been advised of practicing exercise by her oncologist to relieve pain. However, she has not taken his advice because she is afraid of movement and is afraid of her pain might get worse. Her family think that she exaggerates, and somehow, she is afraid that they are right and there is something wrong with her mind. ¿What do you think about what happens to Soraya? Which healthcare professional should she go to? What do you recommend her to overcome this situation?*

**Case 1. Soraya**		
**Competence: dimensions**	Knowledge and behaviours	Achievement assessment:
**Identify, analyse:** identify and analyse the significant elements, summarize and integrate the different parts, organize the elements and the connection among them, deduce some ideas and/or results and provide some conclusions.	Soraya should: 1. *Notice* that her widespread pain is due to her oncologist process, it doesn’t imply necessarily a serious damage. Her pain can be dealt with by different healthcare professionals. 2. *Analyse* and *recognize* the involving factors in perception and perpetuation of her pain. (i.e., catastrophism, hypervigilance, fear-avoidance behaviours). 3. *Know* the different strategies to relieve persistent widespread pain (i.e., graded exposure, therapeutic adherence, etc.).	*Cognitive assessment:* - Revised Neurophysiology of Pain Questionnaire (R-NPQ) - Develop diagrams that correlate features of perceived pain with strategies and follow-up evaluation outcomes. - Rubric for real practical cases.
**Communicate needs:** intonation and volume, level of preparation of exposition, gestures and body language, clarity of exposure, ability to answer questions, speech clarity, structure, and sequence within the speech.	Soraya should: 1. *Use* assertive, structured, and clarified communication with healthcare professionals, where she can express her needs and concerns. 2. *Develop* the ability of expressing her feelings and needs as well as arguing substantially about the acquired knowledge relating her oncological process to enhance her family understanding.	*Cognitive assessment:* 1. Simulation of reasoned presentation of arguments and feelings facing of health professionals and family members. 2. Rubric for practical real cases.
**Making decisions**: apply systematic methods to take decisions, compile and analyse data to take the most suitable decision, show certainty, be consistent with the solution adopted, collaborate with other on taking decisions.	Soraya should: 1. *Define* her objectives and the strategies to reach them. 2. *Request* professional intervention from Physiotherapy, Psychology and Medicine fields. 3. Physiotherapist may help her to relieve her pain and avoid its exacerbation. Moreover he/she can provide strategies to perform therapeutic exercise and deal with pain management. 4. Physiotherapist may refer her to other healthcare professionals, such as psychologist or pain unit of her reference hospital. 5. *Adopt* new behaviours related to health: she would be recommended to practice physical exercise by graded exposure of movement and improve her self-efficacy at including new strategies to better manage her pain.	*Cognitive assessment:* 1. Rubric for real practical cases. *Behavioural assessment:* 1. Fulfil the pain diary using the adopted strategies and perceived effects, as well as the therapeutic exercise adherence.

***Practical case 2.*** *Belén, she has been very frustrated since her last visit to her physical therapist. It turns out that now, after a long time of treatment with those massages and those other things that she/he did for her that were so good for her, she/he has told her that she does not have to go back any more to her/his physiotherapy clinic and that she should be more responsible for her treatment. The table of exercises that she/he has given to her is quite complicated and she does not intend to do it, she just wants to continue to be treated and supported, she feels totally helpless, and to top it off she perceives that she is getting worse. What factors concur in this case to cause this situation? Who do they mainly depend on? How could they be improved?*

**Case 2. Belén**		
**Competence: dimensions**	Knowledge and behaviours	Achievement assessment:
**Identify, analyse**: identify and analyse the significant elements, summarize and integrate the different parts, organize the elements and the connection among them, deduce some ideas and/or results and provide some conclusions.	Belén should: 1. *Explore* the motivations and *analyse* the arguments of the physiotherapist to stop continued treatment, as well as the strengths and weaknesses in her/his behaviour. 2. *Assess* what possible intrinsic factors are involved in exacerbating her pain (i.e. insufficient proactivity and self-efficacy)	*Cognitive assessment:* 1. Structured table to identify the physiotherapist and Belén´s strengths and weaknesses. 2. Rubric for real practical cases.
**Making decisions:** apply systematic methods to take decisions, compile and analyse data to take the most suitable decision, show certainty, be consistent with the solution adopted, collaborate with other on making decisions.	Belén should: 1. *Re-establish* communication with her physiotherapist to convey her disagreement, her concerns, and the questions she needs to understand the professional’s decision and the evolution of her process. 2. *To be* an active part of her therapeutic process assuming responsibility and commitment. She would be encouraged to assess what achievable short-term goals she would like to achieve and to what extent she is willing to get involved.	*Cognitive assessment:* 1. Rubric for real practical cases. *Behavioural assessment:* 1. Every Monday, fulfilment of a commitment document that reflects the specific objectives that she proposes that week, and what measures will be carried out to achieve them.
**Know how to manage:** identify the different resources, access resources, use resources efficiently, assess the suitability of resources based on the results of use.	Belén should: 1. *Identify* what elements make it easier and/or difficult for her to continue with the guidelines and recommendations provided by her physiotherapist. 2. *Manage* their therapeutic process in a strategic way, relying on the favourable factors, and, as far as possible, make use of the resources available to reduce the obstacles identified (i.e., communicate to the physiotherapist her learning preferences to be able to better integrate the prescribed exercises).	*Cognitive assessment:* 1. *Development* of a flow chart that specifies how she is going to manage her therapeutic process based on the facilitating factors and barriers analysed (for example, if pain intensifies after exercise, the pain mitigation and management strategies will be adopted previously agreed with her physiotherapist). 2. Rubric for real practical cases.

***Practical case 3.*** *Marta, once her intervention in the breast and the accompanying treatments had been resolved, has had pain in her shoulder for more than two years to which she was used to and with which she had reached an agreement so that it would not limit too much her life, "what can I do!", "I must continue living! Yesterday, after a slight fall in which his shoulder hit a wall, a different pain appeared, even more intense. As she lives with pain, she has decided not to consult anyone about this new pain, she thinks that it will be handled the same as the one she has suffered for two years. Do you think she is acting correctly? What would you do if you where her?*

**Case 3. Marta**		
**Competence: dimensions**	Knowledge and behaviours	Achievement assessment:
**Identify, analyse:** identify and analyse the significant elements, summarize and integrate the different parts, organize the elements and the connection among them, deduce some ideas and/or results and provide some conclusions.	Marta should: 1. *Know* that there are different processes that occur with pain and not all of them manifest or evolve in the same way, and that, based on one type of pain, different ones may appear, depending on the tissue and/or mechanism involved. 2. *Know* that regardless of the type of pain and its evolution time, there are different ways of approaching and coping that can be used, from different healthcare professionals, to manage and control the perception and perpetuation of pain.	*Cognitive assessment:* 1. Revised Neurophysiology of Pain Questionnaire (R-NPQ) 2. Pain Cards Game: She will be shown cards with short phrases that include random pain features. Marta will have to associate them with the different pain mechanisms. 3. Rubric for real practical cases.
**Problem solving:** define the problem, identify strategies, propose solutions/hypotheses, evaluate potential solutions, implement solutions, evaluate results.	Marta should: 1. Define both types of pain. 2. *Identify* as a strategy that going to health professionals, including a specialized physiotherapist, can help her improve her perception and persistence of pain, whatever the underling mechanism of pain and the time elapsed since its appearance. The physiotherapist could deliver techniques such as therapeutic education and exercise in addition to manual therapy. 3. *Implement* this strategy and assess its results.	*Cognitive assessment*: 1. Structured document to identify positive aspects of change in her therapeutic process. 2. Weekly fulfilment of acquired strategies and perceived effects. 3. Rubric for real practical cases.
